# Lipomatous lesions around the shoulder: Recent experience in a Nigerian hospital

**DOI:** 10.4103/0973-6042.50877

**Published:** 2009

**Authors:** Ganiyu A. Rahman, Adekunle Y. Abdulkadir, I. F. Yusuf

**Affiliations:** Departments of Surgery, Ilorin, Nigeria; 1 Radiology, University of Ilorin Teaching Hospital, Ilorin, Nigeria

**Keywords:** Lipoma, shoulder joint, shoulder functions

## Abstract

We present four cases of shoulder lipomas in two females and two males in their fourth to fifth decades of life. All four lipomas were big. Three were subcutaneous and one was intermuscular. None of them were associated with any functional limitation of the affected shoulder. Subcutaneous or intermuscular lipomas around the shoulder do not appear to affect shoulder functions. Complete surgical excision is rewarding and was achieved under local anesthesia in all our patients with no incidence of recurrence.

## INTRODUCTION

Lipomas are commonly benign and account for almost 50% of all soft tissue tumors. Lipoma can be classified on the basis of their anatomical location, clinical evaluation, or histological findings.[1] Subcutaneous lipomas are the commonest but subfascial (subaponeurotic) and intermuscular variants are also seen. Most lipomas occur in the trunk, head, and neck.[1]

A lipoma typically manifests as a discrete, mobile, palpable, doughy, solitary soft tissue mass. They enlarge slowly and are frequently asymptomatic. Clinical symptoms are uncommon but they may cause local pain and tenderness, limitation of the range of motion of a joint, and nerve compression.[2] Superficial lipomas can be accurately diagnosed on the basis of clinical findings in up to 85% of cases.[3,4] A diagnostic test used by some clinicians for superficial lesions is a hardening of the mass after application of ice.[5]

Lipomas may increase in size during periods of rapid weight gain. The converse is however not true during periods of severe weight loss, at which time these lesions may become more apparent because the fat in lipomas is unavailable for general metabolism.[5] Deep lesions may also become more apparent as distinct masses during muscle contraction.

Superficial lipomas rarely present with symptoms other than a visible swelling. The diagnosis is generally made on clinical grounds, without recourse to radiological workup. Surgical excision is the treatment of choice.

We present this case series of shoulder lipomas seen in our practice. The emphasis is on the presentation, radiographic findings, treatment, and outcome of treatment.

## CASE REPORT

### Case 1

**Figure 1:**
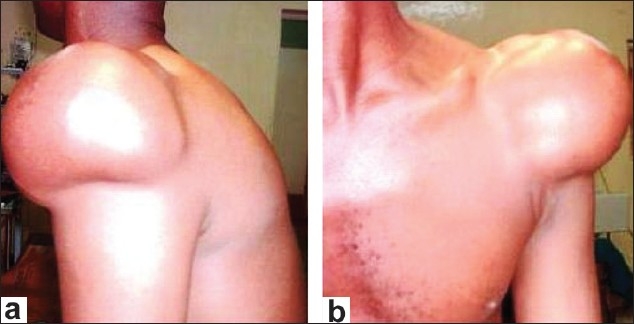
Photograph showing a subcutaneous left shoulder lipoma appearing as a 'cap' on the deltoid

A 33-year-old man presented with 5 years' history of a gradually increasing, painless, left shoulder swelling. There was no associated fever or weight loss. There was a preceding history of minor trauma to the affected shoulder. Examination revealed a circumscribed left shoulder swelling of about 14 × 12 cm, with positive lobulation and slipping signs [Figure 1]. There was no limitation of movement at the joint. The patient's weight was 64 kg and he was 1.68 m tall (Body Mass Index (BMI) = 22.7). The clinical examination findings suggested the diagnosis of subcutaneous lipoma of the left shoulder. Plain radiography demonstrated a soft tissue mass of lower density than the surrounding soft tissue; there was no calcification within the mass. The underlying bones were normal. The patient's outcome was satisfactory following excision of the mass under local anesthesia. At surgery there was a well-encapsulated subcutaneous fatty mass of about 14 cm × 12 cm × 8 cm size that weighed 800 gm. Histology showed fibrolipoma. Three and a half years post excision there is no clinical sign of recurrence.

### Case 2

A 30-year-old woman presented with a 7-year history of a slowly growing painless mass over the right shoulder. There was no history of any trauma or of weight loss. On examination, a firm, nontender, well-circumscribed, mobile mass was found over the right shoulder, extending to the area over the right trapezius. Her vital signs and shoulder movement were essentially normal. We arrived at a clinical diagnosis of subcutaneous lipoma and the mass was excised. We did not do a radiological evaluation prior to excision. At surgery, done under local anesthesia, there was a well-encapsulated, subcutaneous fatty mass of 7 cm × 6 cm × 6 cm size. Histology confirmed lipoma. She has been followed up for 3 years post operation and has had no recurrence.

### Case 3

**Figure 2:**
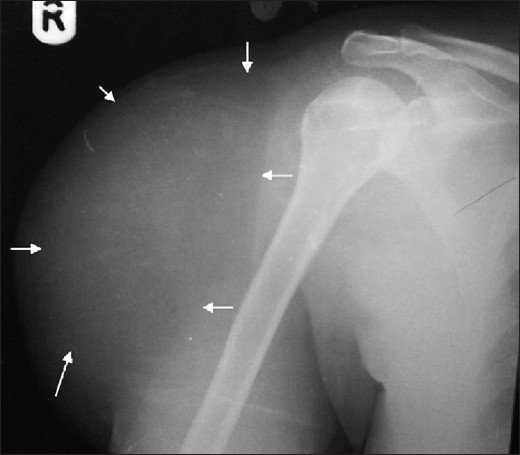
Radiograph showing a subcutaneous lipoma over the right deltoid. Note its well-defined plain (arrow) and the relative radiolucency as compared to the adjacent soft tissue

A 41-year-old woman with a history of asthma since childhood and 8 years' history of hypertension presented with a 3-year history of slow-growing and painless swellings over the right shoulder and the anterior abdominal wall. She was more concerned about the swelling on the anterior abdominal wall because it was hard in consistency. This was excised under local anesthesia and histology showed it to be a fibroma. The right shoulder mass, measuring 26 cm × 26 cm × 12 cm by clinical estimation, had visible superficial veins and was firm in consistency, nontender, well-circumscribed, and lobulated. There was no limitation of movement of the joint. On the basis of the clinical findings, we made a diagnosis of subcutaneous lipoma. However, we also kept in mind the possibility of a soft tissue sarcoma. A plain radiograph of the shoulder demonstrated a lucent soft tissue mass over the deltoid with well-defined planes [Figure 2]. After her high blood pressure was controlled, she had an uneventful excision of the right shoulder mass under local anesthesia. At surgery, the mass was found to be highly vascular, with the presence of a pseudocapsule. There was no evidence of invasion of the adjacent muscles but there was extension between the fibres of the deltoid and biceps muscles (intermuscular). It weighed about 950 g. Histology showed lipoma. The patient is normal, without recurrence, 28 months after surgery.

### Case 4

A 35-year-old woman presented with a circumscribed, mobile, firm, and painless swelling of 5 cm × 3 cm × 2 cm size over the anterior aspect of the right shoulder. There was no enlargement of the regional lymph nodes. The functioning of the shoulder joint was not affected. A diagnosis of subcutaneous lipoma was made. She had surgical excision under local anesthesia and histology confirmed lipoma. The patient has been followed up for 30 months and there has been no evidence of recurrence.

## DISCUSSION

Lipoma is the commonest soft tissue tumor and may be found in any part of the body. Benign lipomatous lesions are common in both soft tissue and in bone. The prevalence of soft tissue lipoma has been estimated at 2.1 per 100 persons.[4,6‐8] In 2002, the World Health Organization Committee for the Classification of Soft Tissue Tumors categorized benign lipomatous lesions involving the soft tissues into nine entities: lipoma, lipomatosis, lipomatosis of nerve, lipoblastoma/lipoblastomatosis, angiolipoma, myolipoma of soft tissue, chondroid lipoma, spindle cell/pleomorphic lipoma, and hibernoma.[3]

It is unclear if a soft tissue lipoma represents a benign neoplasm, a local hyperplasia of fat cells, or a combination of both processes. Lipomas can be superficial or deep and commonly affect the upper back, neck, proximal extremities (particularly the shoulder), and abdomen.[5] Lipomas may be multiple in 5–15% of patients.[1,3,6] Multiple lipomas tend to be commoner in males (M:F ratio of 6.6:1); familial in approximately 30% of cases; predominate in the back, shoulder, and upper arms; may be symmetric; show a predilection for the extensor surface; and are most common in the fifth to sixth decades of life.[1,3,6,7] In 1992, Enzi *et al*.[9] reported six cases of shoulder girdle lipomatosis. All were in women between the ages of 38 and 72 years. In our case series the male:female ratio was 1:1 and the patients were between the ages of 30 and 41 years. Lipomas most commonly occur in the fifth to seventh decades of life, with 80% of lesions found in patients 25–85 years of age.[3,6] No patient in our series had multiple lipomas.

Lipomas around the shoulder are known to infiltrate between the muscles of the extremities and the thoracic wall.[9] Most of the cases seen in our series were subcutaneous, and only one was intramuscular. Superficial lipomas are smaller than 5 cm in 80% of reported cases and only 1% is greater than 10 cm in size.[6] Again, this is at variance with our findings; two of our four patients had shoulder lipomas greater than 10 cm in at least two of the three dimensions of measurements. According to Rydholm and Berg,[6] such large lipomas occur because patients generally ignore them in their early stages and most patients have had the lesions for more than 10 years before seeking medical care. All our patients were asymptomatic and had no limitation of the range of movement of the shoulder in any plane. A superficial lipoma may cause nerve compression in approximately 25% of patients.[2]

The lipomas seen in our patients typically presented as discrete, mobile, solitary soft tissue masses. All of them were otherwise asymptomatic. This is unlike the findings of Regan *et al*.[2] who reported associated clinical symptoms in 25% of patients. Most patients with superficial lipomas in our series were diagnosed by clinical evaluation. However, in deep lipomas, this may not be possible, as clinical evaluation will indicate only a nonspecific mass. Therefore, deep lipomas may frequently require imaging.

Small lipomas often may not be noticeable on radiography, while larger lipomas may show a typical radiolucency [Figure 2]. Underlying osseous abnormalities are rare. We too found no osseous involvement in our patients.

The sonographic appearance of a lipoma is that of a hyperechoic mass having no posterior acoustic enhancement.[6,10] This intrinsic echogenicity makes distinction of the echogenic capsule difficult. Heterogeneity caused by septa or other nonlipomatous components may be identified. The fat in lipomas, which is a hindrance to sonographic evaluation, works as an advantage in CT or MR imaging, both of which are superior to ultrasonography for the confident identification of adipose tissue in these lesions.

Surgical excision is still the best form of treatment. We have found it to be very rewarding, providing 100% cure in all our patients. The lipomas in this series were excised en bloc under local anesthesia as day cases to save cost and patient time. All our cases were lipomas or fibrolipomas and there was no evidence of sarcoma in any of them.

## CONCLUSIONS

Our case series includes lipomas around the shoulder; the majority was subcutaneous and there was no functional limitation of the affected shoulder in any of the patients. Thus, lipomas around the shoulder joint, whether subcutaneous or intramuscular, do not seem to affect shoulder function. Complete surgical excision is rewarding and was achieved under local anesthesia in all our patients with no incidence of recurrence.
